# Influence of the ultra-fine fly ash dosages on the mechanical properties of the sulfoaluminate cement-based high water backfilling material

**DOI:** 10.1038/s41598-022-23547-5

**Published:** 2023-01-28

**Authors:** Yaohui Sun, Shengrong Xie, Chaowen Wu, Junqi Cui, Dongdong Chen, Fangfang Guo, Zaisheng Jiang, Yuxin Ren, Weiyong Lu

**Affiliations:** 1 Department of Mining Engineering, Lyuliang University, Shanxi, 033001 China; 2 School of Energy and Mining Engineering, China University of Mining and Technology (Beijing), Beijing, 100083 China

**Keywords:** Solid Earth sciences, Chemistry, Engineering, Materials science

## Abstract

To reduce the filling cost of high-water backfilling material (HWBM) in mining backfill and improve the recycling utilization of the industrial waste such as the coal fly ash. The ultra-fine fly ash (UFA) was added to the HWBM as a partial replacement in this work. Therefore, a series of experiments were performed to investigate the effect of UFA on the mechanical properties of the HWBM at the different curing conditions, then the hydration mechanism of the HWBM blended with UFA was analyzed by XRD and SEM method. The result indicates that the strength of the HWBM decreased with the increasing of UFA dosages, but the addition of UFA can improve the residual strength of the initial HWBM. Additionally, when the HWBM was cured at the laboratory air condition, its carbonation process was restrained obviously as the UFA dosages were less than 15% at the ages of 28 days, which indicates the UFA can improve the weathering resistance of the HWBM with the curing ages increasing effectively. The XRD and SEM results also shows that the degree of crystallinity of the HWBM increased when UFA dosages were less than 15% effectively, while there were few obvious changes on types of hydration products. It indicates that the main affects of UFA on the performance of HWBM is filler and dilution, which reduced the contact area between hydration products of HWBM and CO_2_ in the air, further improved the carbonation resistance of HWBM.

## Introduction

The high water backfill material (HWBM) is an excellent mine backfill material which has the advantages of good liquidity, short initial setting time, and good early strength, it consists of material A and material B, which the material A is compound of calcium sulfoaluminate (CSA) cements, retarder, and suspension agent, while the material B is compound of quicklime, anhydrite, accelerator, and suspension agent. Two types of slurry would not solidify when they were mixed and stirred with water within 24 h, but the slurry would solidify rapidly after two slurries mixed within 30 min. However, the filling cost and strength of HWBM will increase with a decreasing the water cement ratio (W/C), while the strength will decrease with an increasing the W/C^1^. In addition, the disadvantage of poor toughness and small compressibility also limits the application of HWBMs for use as mine backfilling materials^2,3^.

In recent years, for enhancing the performances of HWBMs and reducing the backfill cost, numerous studies have been performed. On the one hand, for reducing the consumption of the cement, amounts of experiments were carried out to improving the water binder ratio (W/B), finally the superhigh water material with the W/C of 6:1–11:1 was researched and developed^2,3^. And the optimal gypsum quicklime ratio in the materials B of the HWBM was be determined from the 8:2 to 8.5:1.5^4,5^. Additionally, the effects of various additives (such as fly ash, gravel, river sludge, lithium carbonate, and aluminum sulfate) on the physical and chemical properties of HWBMs has been investigated^5–10^. These research show that the mechanical properties of the HWBM decreased generally after adding the industrial solid waste, while the mining backfill cost was reduced^6–8^. However, the addition of lithium carbonate and aluminum sulfate can promote the hydration process and setting time of the HWBM^5,9^. Thus, the mechanical properties of the HWBMs should be further improved to adapt the roof settling in mine by adjusting the component of the HWBMs and solid wastes^11–16^.

Whereas fly ash is a well solid waste and generally is used in cement-based composite material as a supplementary cementing material^17–19^, which there are sufficient resources in China mining. Thus, the fly ash also has been application on many engineering filed, such as building concrete material, mining cement-based grouts material, and mining backfill material,etc^20–23^. Additionally, for improving the activation of fly ash, ultra-fine fly ash was studied to modify the hydration process and mechanical properties of the cement composite materials, which the research found that the UFA can contribute better workability and compressive strength of 28 days than FA.

Meanwhile, expect for the silicate cement-based mining backfill materials, the application of the fly ash on the HWBMs also have been researched in recent years^11,15,18^. In these research, amounts of experiments has been carried out to study the addition of fly ash on the hydrated heat, early strength, setting time and hydration products of the HWBM. The research found that the addition of fly ash can reduce the hydrated heat and enhance rheological behavior and residual strength, while the backfill cost was reduced of the HWBM. But the uniaxial compressive strength, elastic modulus, and deformation modulus of HWBM all decreased with the fly ash dosages increasing due to that the addition of fly ash consumed the CH in the HWBM at the early ages.

However, there are limited experimental words reported on the influence of UFA on the mechanical properties and durability under air environment of the high water backfill materials. Hence, the objection of this paper is to investigate the effects of fly ash on the mechanical properties of HWBM, then explore the hydration reaction mechanism between fly ash and the HWBM curing at different environment.

Therefore, main objective of this work is to evaluate the effect of the fly ash on the mechanical properties of the HWBM under the different curing conditions and curing ages. In addition, the influence of the fly ash dosages on the modification efficiency of the HWBM also be investigated. Meanwhile, the X-ray diffraction (XRD) and scanning electron microscope (SEM) tests were carried out to investigate the effects of UFA on the hydration products and microstructure of the HWBM, further analyze the hydration process of the HWBM after blended with the UFA. Additionally, the effects of UFA on the performance of the HWBM at the weathering environment were investigated, and the weathering mechanism of the HWBM was explored.

## Materials and methods

### Materials

**Figure 1:**
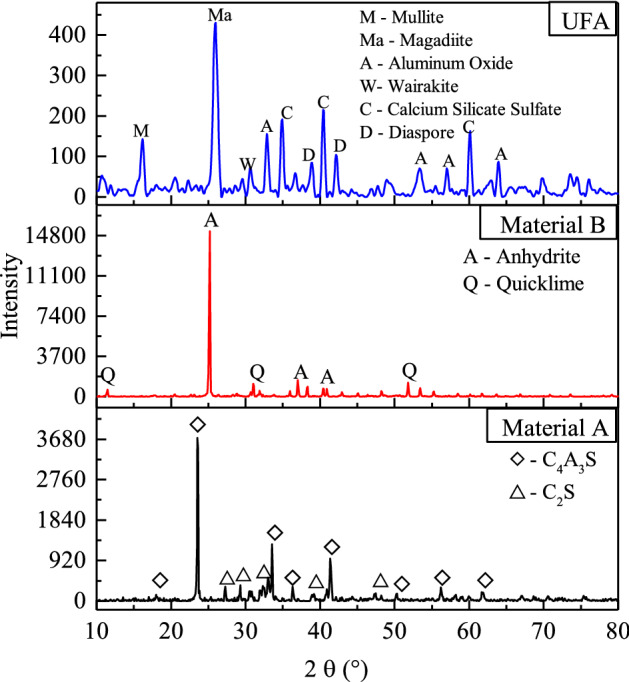
The X-diffraction pattern of the fly ash.

**Figure 2:**
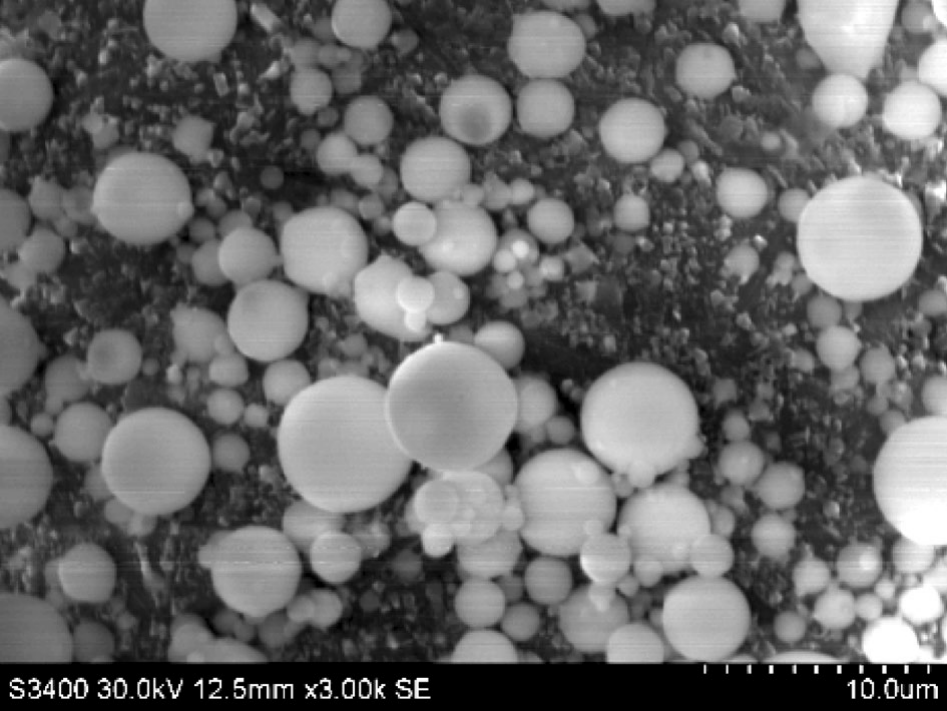
The SEM image of the ultra-fine fly ash.

**Figure 3:**
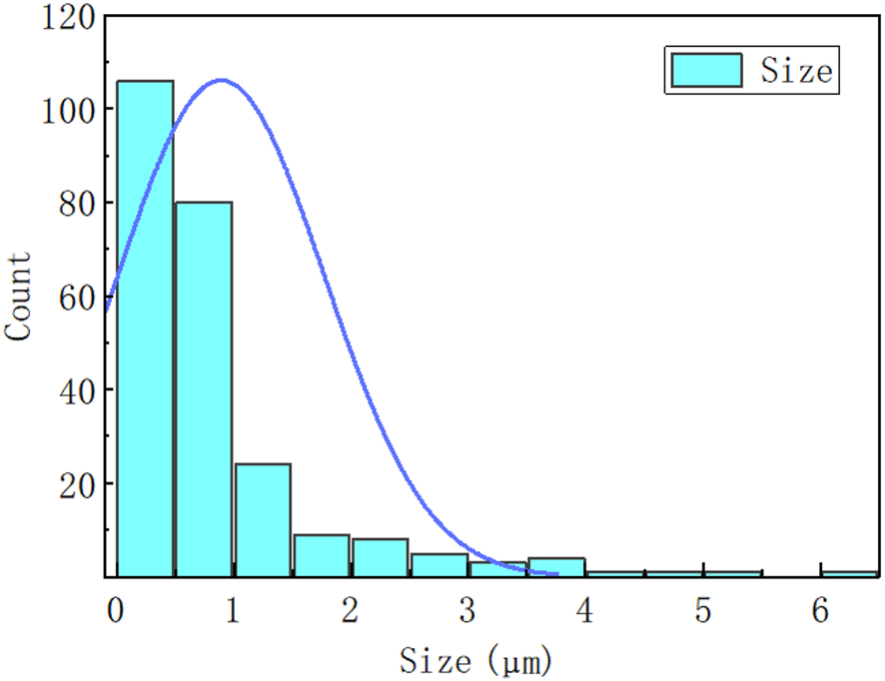
Particle size distribution of ultra-fine fly ash.

The HWBM comprise material A and material B, which the material A is compound of calcium sulfoaluminate (CSA) cements, retarder, and suspension agent, while the material B is compound of quicklime, anhydrite, accelerator, and suspension agent. Additionally, in this work, the fly ash was sampled from a China Henan thermoelectric power plant that burned coal as fuel. The chemically composition and microstructure of the fly ash was determined by XRD and SEM as shown in Fig. 1. The XRD pattern displays that the mineralogical composition of the UFA. Meanwhile, as shown in Fig. 2, the SEM image of UFA shows that the UFA particle vitric, and its size are all less than 5 μm by statistical treatment used in Image J software as shown in Fig. 3. And the chemical composition of the HWBM and UFA are shown in Table 1.

**Table 1:** Chemical composition of the material A, B and fly ash/%.

Compounds	Al_2_O_3_	SiO_2_	CaO	FeO	MgO	SO_3_	Loss
Material A	33.12	7.81	44.95	2.16	0.69	9.01	2.26
Material B	1.46	3.06	34.38	1.36	3.02	54.22	2.5
UFA	37.7	45.1	6.45	1.85	1.2	3.1	4.6

### Methods

#### Preparation of HWBM samples

In this work, the “blend” refers to that the UFA is as a partial substitute of material A and material B in HWBM, and “dosages of UFA” refers to the “substitutionary quality of UFA in HWBM”. A constant water solid ratio of 2.5 was used to investigate the effect of the UFA content on the performance of HWBM. The dosage of UFA in slurry A and B of HWBM was 0, 5, 10 15, 20, 30 wt.% by mass. The well mixed paste slurries were cast in the moulds of φ50 mm × 100 mm with a primary cure for 2 h. After demould, half of specimens were sealed by plastic film and further cured for 1 day, 3 days, 7 days, 14 days, 28 days at a temperature of 20 °C and a relative humidity ≥ 95%, while half of specimens were cured under the laboratory air condition at 20 ± 5 ℃ and 45 ± 5 ℃ relative humidity. Later, the FA-HWBM specimens were subjected to a drying treatment at 90 °C to remove the water confined in the pores and/or adsorbed on the pore surfaces.

#### Uniaxial compression strength tests

The compression strength of the HWBM samples was measured using a WAW-600B electrohydraulic servo universal testing machine (Tianshui Hongshan Testing Machine Co, Ltd, China). A loading rate of 2 mm/min was continually applied by controlling the axial strain until the specimen failed. Additionally, the elastic modulus and deformation modulus of the samples was calculated according to the stress–strain curves.

#### XRD and SEM testis

Before the XRD and SEM tests, it was important to seal the samples before the XRD and SEM analysis, to prevent the process of sample carbonation that occurs when the samples contact with CO_2_ present in the atmosphere, and the samples were first dried under 50 ℃ for 48 h for ending the hydrate process of samples. Then the X-ray diffractometer (PANalytical Corporation) was used to investigate the mineralogical composition of the HWBM with different dosages of UFA curing for 7 days and 28 days. During the test process, 2-theta degree ranged from 10°to 45°. Then the MDI Jade 6.0 software was used to obtain the mineralogical phase of the HWBM samples. Additionally, SEM was used to observe the effects of the UFA dosages on the surface morphologies of the HWBM samples.

## Results and analysis

### The setting time of the HWBMs blended with UFA

**Figure 4:**
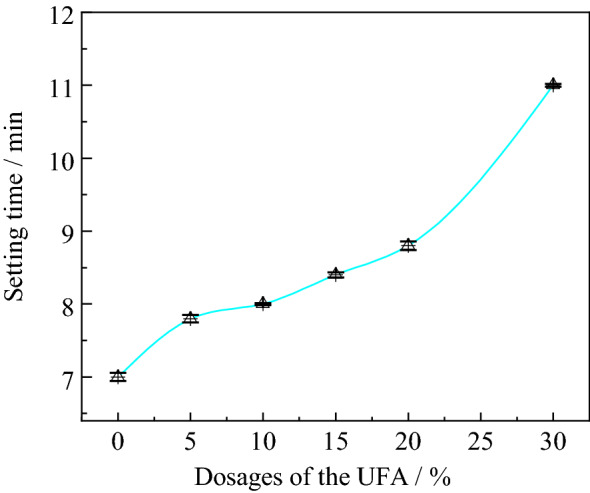
Setting time of the HWBM blended with the UFA.

According to the coal industrial standard (MT/T420-1995) of the HWBMs in China, the setting time of the HWBMs should be less than 20 min. In this work, the setting time of the HWBMs was 7 min, which is up to standard as shown in Fig. 4. The setting time of the HWBMs increased with the increasing of the UFA dosages. And particularly, the setting time of the HWBMs blended with the UFA increased rapidly as the UFA dosages increased from 20 to 30%. However, the setting time of the HWBMs blended with the UFA of 30% still did not exceed 20 min. It indicates that the addition of the UFA delayed the hydration process of the HWBMs, and the 20% is a inflection point, which the setting time of the samples increasing rapidly as the UFA dosages exceeded 20%.

### Mechanical properties of the HWBMs blended with UFA

#### Mechanical properties of the HWBM cured at the standard curing condition

At the standard curing condition, the stress–strain curves of the HWBM blended with UFA of different dosages were tested shown, and the change in the UCS, elastic modulus and deformation modulus of the HWBM with different UFA dosages were tested and calculated for analyzed the influence of the UFA on the mechanical properties of the HWBM.

**Figure 5:**
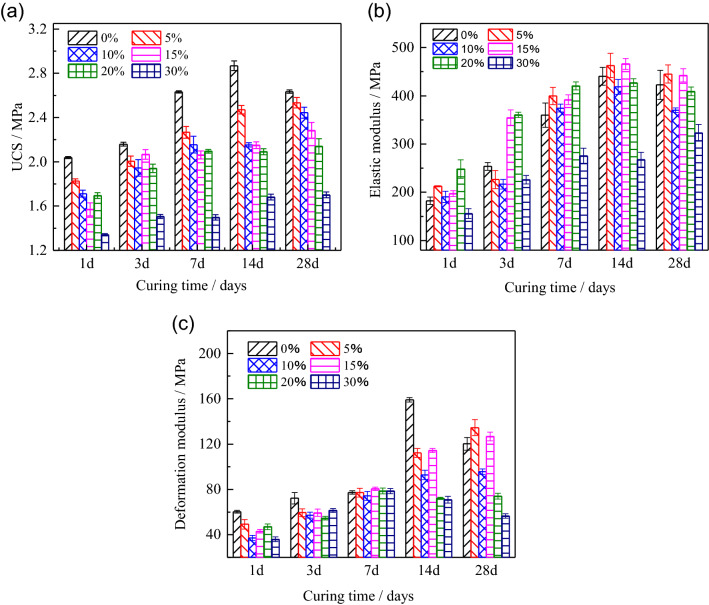
The mechanical properties of the HWBM with different UFA dosages and curing times at the standard curing condition: (**a**) UCS; (**b**) elastic modulus; (**c**) deformation modulus.

Under the standard curing condition, the strength, elastic modulus, and deformation modulus of the HWBM blended with the UFA of different dosages were calculated in Fig. 5. Firstly, as shown in Fig. 5a, it can be observed that the strength of the HWBM decreased with the increasing of the UFA dosage at different curing ages (1, 3, 7, 14 and 28 days), and the strength of the HWBM show a sharp decreased when the dosage of UFA was 30%, this result is similar with previous study^5^. However, with the increasing of the curing time, the later strength (at the age of 28 days) of the initial HWBM decreased by 8.15%, but the strength of HWBM blended with UFA maintained increasing at the later age. It indicates that the addition of UFA has a positive influence on the increasing of strength at the later age.

Meanwhile. Elastic modulus (E) is an important performance parameter to evaluate the capacity of resistance to elastic deformation of backfill body in mine backfill field, which can reflect the elastic deformation capacity of HWBM under the roof pressure. As shown in Fig. 5b, there are same change trend in E and UCS of initial HWBM, which indicates there are a few limitations on elastic deformation capacity of pure HWBM. However, after blended with UFA, the elastic modulus of the HWBM have slightly increased when the dosages were 5% and 15%, but with the elastic modulus decreased sharply with the dosage increasing to 30%.

Additionally, the deformation modulus of HWBM also is an important parameter to evaluate the deformation capacity and compressibility of backfill body. As shown in Fig. 5c, the deformation modulus of the HWBM first increased and then decreased, while the peak occurred at the age of 14 days. After addition of UFA, the deformation modulus increased with curing time increasing. It indicates that the addition of UFA has a positive effect on deformation modulus of the HWBM at the later ages.

In summary, there are a few limitations on later mechanical properties of pure HWBM, but the addition of UFA has a positive effect on these limitations. However, with the UFA dosages increasing, the mechanical properties of the HWBM blended with UFA still decreased comparing with pure HWBM, especially the mechanical properties decreased sharply when the UFA dosage was 30%. Thus, we suggest that the UFA dosage should be less than 30% in mining backfill engineering.

**Figure 6:**
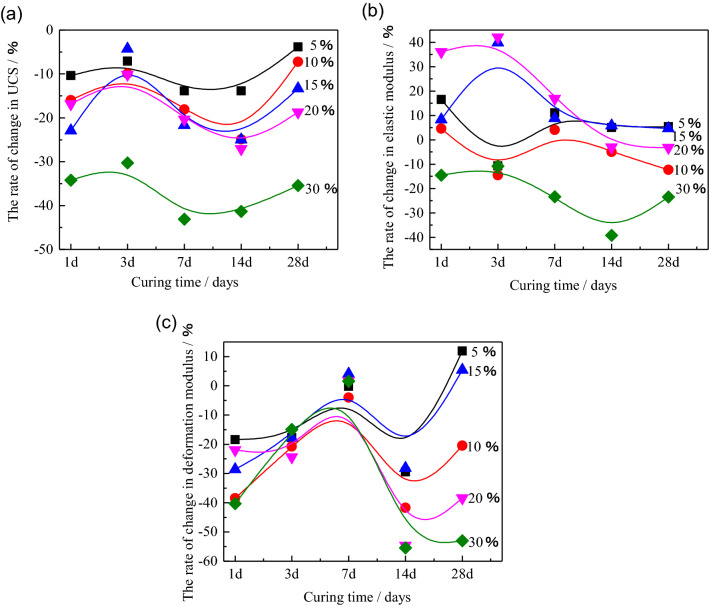
The variation rate of the UCS, elastic modulus and deformation modulus of the HWBM with different UFA contents compared with the control group at the standard condition. (**a**) UCS; (**b**) elastic modulus; (**c**) deformation modulus.

Moreover, for further investigating the change rate of the HWBM blended with UFA comparing with the initial HWBM, the variation rate of the UCS, elastic modulus and deformation modulus of the HWBM with different UFA contents compared with the control group at the standard condition were calculated in Fig. 6.

Figure 6 displays the variation rate of the UCS, elastic modulus and deformation modulus of the HWBM with different UFA contents compared with the control group at the standard condition for further analyzing the variation rate of the mechanical properties of the HWBM blended with UFA of different dosages. It can be observed that the gap of the mechanical properties between the pure HWBM and the HWBM blended with UFA reduced with the curing time increasing under the standard curing condition, while the strength decreasing by less than 10% as the UFA dosages less than 10%. Moreover, the E and D have increased to some extent when the UFA dosages are 5% and 10%, which indicates that the a few additions of UFA have a positive influence on the development of mechanical properties of the HWBM at the later ages.

**Figure 7:**
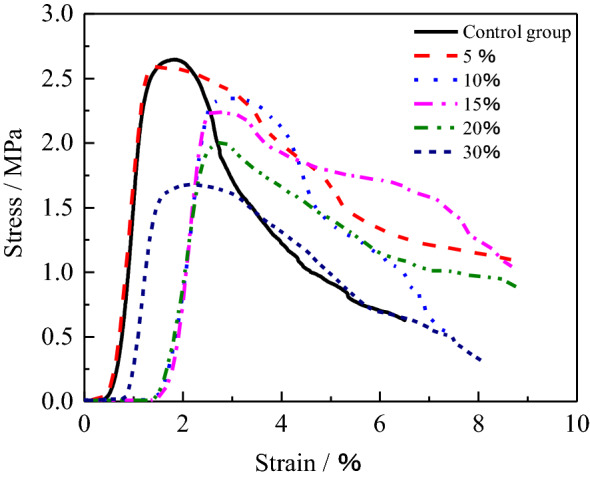
The stress–strain curves of the HWBM mixed with different content UFA at the standard curing condition curing for 28 days.

For analyze the deformation characteristic of the HWBM with the UFA of different dosages, the stress–strain curves of the samples after curing for 28 days at the standard curing condition were shown in Fig. 7. It can be observed that the strain of compaction stage of the initial HWBM is shorter than the samples blended with UFA, and quickly enter elastic stage, then the strain is lesser when attained peak stress, while the strain at the peak stress increased with UFA dosages increasing. It indicates that the initial HWBM can realize support of the roof as the stain is lesser, However, after mixed with UFA, the HWBMs still have higher strength after attained peak strength, while the stress of initial HWBM decreased quickly. It indicates that the UFA can enhance the residual strength of the HWBM, which makes the HWBM has higher stress to support the roof deformation and ground surface settlement.

#### Mechanical properties of the HWBM cured at the laboratory air condition

Comparing with the wet and closed environment in coal goal, the dry air and high-temperature environment in roadway has a obviously negative affect on the mechanical properties of the HWBM. The previous reports^3^ find that the strength of superhigh water material (water content attains 95–97%) decreased with the increasing of curing ages in weathering condition and lost its stability after cured for 28 days entirely. Therefore, it is necessary to investigate the effect of the UFA on mechanical properties of the HWBM at the weathering environment. In this work, the laboratory air condition was used to investigate the change in the mechanical properties and deformation characteristics of the HWBM as the simulation of the coal roadway.

**Figure 8:**
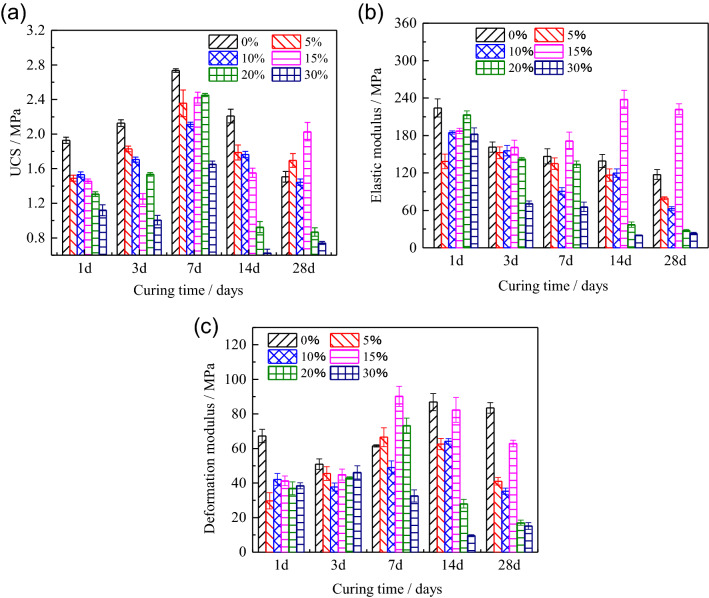
The mechanical properties of the HWBM with different UFA dosages and curing times at the laboratory air condition: (**a**) UCS; (**b**) elastic modulus; (**c**) deformation modulus.

Figure 8a shows the UCS of the HWBM blended with different dosages of UFA at the ages of 1, 3, 7, 14 and 28 days cured at the laboratory air condition. It shows that the strength of the initial HWBM first increased and then decreased with the increasing of curing time, while the peak strength occurs at the age of 7 days. However, after blending with UFA, the 28 days strength was higher than the initial HWBM when the dosages of UFA were less than 15%. Nonetheless, the strength of the HWBM reduce sharply after ages of the 14 days when the dosages were 20% and 30%, this result suggest that the UFA dosage should be less than 20% when the HWBM is applied in the gob-side entry retaining of mining engineering.

Meanwhile, elastic modulus of the HWBM blend with different dosages of UFA at different curing ages were calculated in Fig. 8b. It can be found that the E of initial sample decreased with curing time increasing, while the E of the HWBM showed the trend of first decreasing, then increasing and final decreasing with UFA dosages increasing. It is noteworthy that the E of the HWBM is higher the initial sample by 17.05%, 70.81% and 89.39% respectively at the ages of 7, 14 and 28 days when the dosage of UFA was 15%, which indicates the capacity of resistance to elastic deformation increased significantly in the HWBM blended with UFA of 15% at the later ages. In another aspect, when the UFA dosages exceeded 20%, the elastic modulus of the HWBM decreased sharply at the later ages.

Deformation modulus of the HWBM is shown in Fig. 8c. The Deformation modulus of HWBM first increased and then decreased with the curing time increasing, while the deformation modulus of the samples blended with UFA decreased at the early ages (1 and 3 days). It indicates that the brittle deformation of HWBM increased, which means compressibility of the HWBM decreased after blended with UFA. Particularly, when the UFA dosages were 20% and 30%, the deformation modulus of the HWBM decreased sharply at the later ages. However, when the UFA dosage was 15%, the change in deformation modulus of the HWBM is similar with initial HBWM. It indicates that the effect of UFA of 15% on compressibility of the HWBM is minimum at the laboratory air condition.

**Figure 9:**
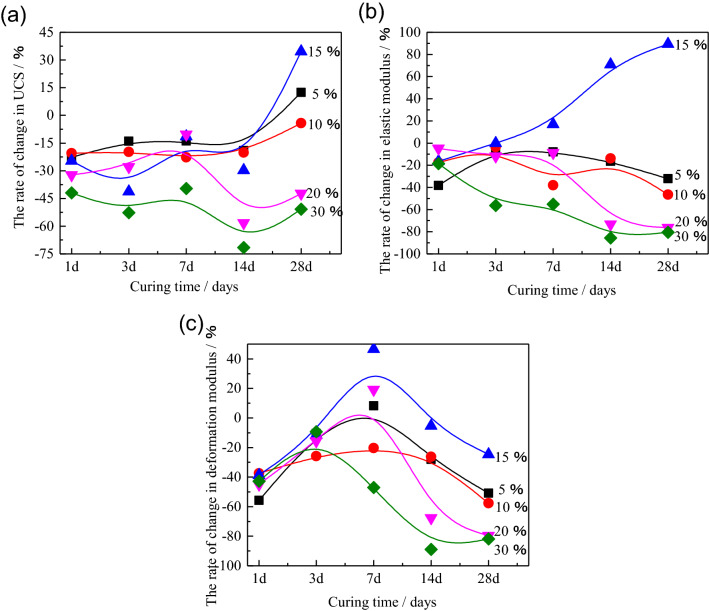
The variation rate of the UCS, elastic modulus and deformation modulus of the HWBM with different UFA contents compared with the control group at the laboratory air condition. (**a**) UCS; (**b**) elastic modulus; (**c**) deformation modulus.

Meanwhile, Fig. 9 is displayed for analyzing the effect of UFA dosage on variation rate of mechanical properties. It can be inferred that UFA dosages of 20% and 30% have a significant weakening effect on the development of mechanical properties of the HWBM UCS of the HWBM with curing time increasing under the laboratory air condition. However, when the dosage of UFA was 15%, the UCS and elastic modulus of the HWBM increased significantly at the later ages, while the deformation modulus decreased with the increasing of curing ages. This result suggests that the 15% is an optimal UFA dosage of HWBM with the water solid rate of 2.5:1 for reducing backfill cost and increasing mechanical properties of the HWBM.

**Figure 10:**
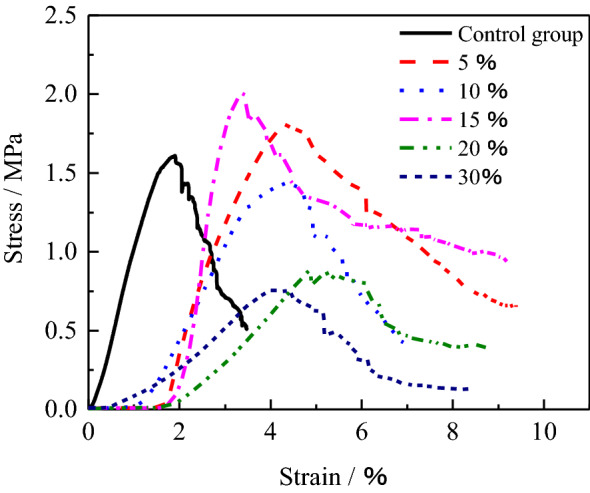
The stress–strain curves of the HWBM mixed with different content UFA at the laboratory air condition curing for 28 days.

In addition, for further exploring the influence of the UFA dosage on the mechanical properties and deformation characteristics of the HWBM, the stress–strain curves of the HWBM with different UFA dosages at the laboratory air condition for 28 days are analyzed. It can be observed that the strength decreased, and deformation increased significantly when the UFA dosages were 20% and 30%, which indicates that UFA dosage of exceeding 20% have an obvious negative effect on the mechanical properties of the HWBM under open dry environment. However, the mechanical properties of the HWBM increased in a deep when the UFA dosage was less than 15%, and the HWBM still has higher strength comparing with the initial HWBM with the strain increasing as shown in Fig. 10. It indicates that the addition of small amount of UFA can reduce the weathering depth of the HWBM effectively under the laboratory air condition.

### Effect of UFA on the hydration products of the HWBM

To investigate the effect of UFA on the hydration process and products of HWBM, the XRD tests were performed to analyze the variation of mineralogical composition in the HWBM with different dosages of UFA.

**Figure 11:**
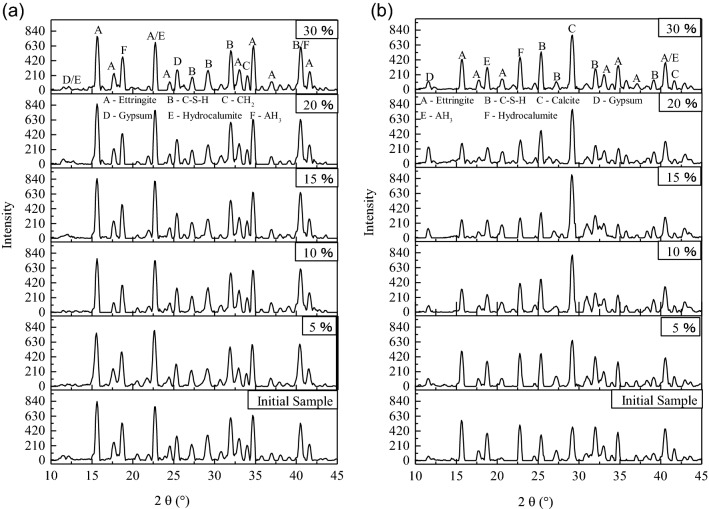
The XRD pattern of the HWBM blended with different contents of UFA cured for 7 days at different curing conditions. (**a**) The standard curing condition; (**b**) the laboratory air condition.

Figure 11 shows the XRD pattern of the HWBM blended with different contents of UFA cured for 7 days at different curing conditions, it can be observed that the main hydration products are ettringite, and a few C–S–H and AH_3_ gel as shown in Eqs. (1)– (3). Additionally, there are small amount of CH_2_ and gypsum. It is noteworthy that the UFA particles have no significant diffraction peaks, which may be the result of too small particle size of UFA and covering by other hydration products structure.

1 $${\text{C}}_{{4}} {\text{A}}{}_{{3}}\overline{{\text{S}}} + 2{\text{CaSO}}_{4}^{{}} + 38{\text{H}}_{{2}} {\text{O}} \to {\text{C}}_{{2}} {\text{A}} \cdot 3{\text{CaSO}}{}_{4} \cdot 32{\text{H}}_{{2}} {\text{O}} + 2{\text{AH}}_{3} (gel)$$

2 $${\text{C}}_{{2}} {\text{S}} + 2{\text{H}}_{{2}} {\text{O}} \to {\text{C - S - H}} + {\text{Ca}}({\text{OH}})_{2}$$

3 $$3{\text{Ca}}({\text{OH}})_{2} + 3{\text{CaSO}}_{4} + {\text{AH}}_{3}^{{}} + 26{\text{H}}_{2} {\text{O}} \to {\text{C}}_{2} {\text{A}} \cdot 3{\text{CaSO}}_{4} \cdot 32{\text{H}}_{2} {\text{O}}$$

As shown in Fig. 11a, it can be observed that the types of hydration products in the HWBM have no obviously changes after blended with different dosages of UFA when the sample were cured at the standard curing condition, which indicates that there are other reactions occurred in the HWBM at the early ages after addition of UFA. This result is consistent with previous study^17^. Hence, it can be inferred that the main effects of UFA addition are filler and dilution at the early hydration process, which caused the mechanical properties of the HWBM decreasing at the early ages, and the strength of the HWBM decreased with UFA dosages increasing. Particularly, the mechanical properties of the HWBM decreased sharply when the UFA dosages exceed 20%.

However, when the HWBMs were cured at the laboratory air condition, the hydration products have obvious changes as shown in Fig. 11b. It can be observed that the diffraction peak of ettringite decreased with the UFA dosages decreasing while there was calcite formed due to the carbonation of ettringite under the open dry environment. However, it is noteworthy that the ettringite content increased when the UFA dosage was 30%, which may be the result of enough UFA filled in the pore of ettringite and delayed its carbonation. Whereas overmuch UFA also destroyed the skeleton structure of ettringite in the HWBM, caused the mechanical properties of the HWBM decreased.

**Figure 12:**
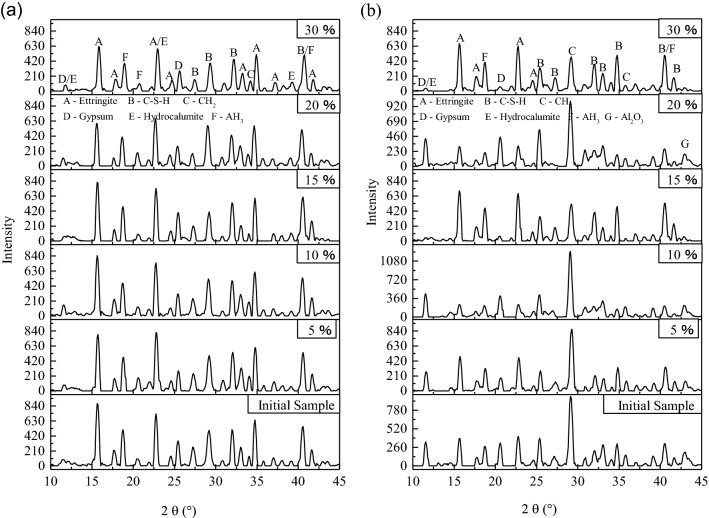
The XRD pattern of the HWBM blended with different contents of UFA cured for 28 days at different curing conditions. (**a**) The standard curing condition; (**b**) the laboratory air condition.

Moreover, for further investigating the change in hydration products of the HWBM with curing time increasing, the XRD patterns were displayed in Fig. 12.

Compared with the XRD patterns of samples at the age of 7 days in Fig. 11a, the types of hydration products have no obviously changes at the ages of 28 days as shown in Fig. 12a. Whereas the diffraction peak of ettringite decreased significantly when the UFA dosages exceeded 20%, it indicates that the overmuch UFA addition have a negative influence on the hydration process of the HWBM, which caused the mechanical properties of the HWBM decreased sharply as shown in Fig. 5a.

On the other hand, when the HWBMs were cured at the laboratory air condition, there are obvious variations in the hydration products of the HWBM with the increasing of the curing ages and UFA dosages. As shown in Fig. 12b, after cured for 28 days, the diffraction peak of the ettringite and C–S–H further decreased while diffraction peak of the calcite further increased compared with the sample cured for 7 days, it indicates that the evaporation of free water accelerated carbonation of ettringite due to the increasing of porosity and contact area between ettringite and CO_2_ in the air. Whereas, after blended with UFA, UFA particles filled into the pore of the HWBM as fillers, caused the decreasing of contact area between hydration products and air, which restrained the carbonation of ettringite. Thus, the strength of the HWBM increased at the ages of 28 days when the UFA dosages were less than 15% as shown in Fig. 8a, but when UFA dosages were 20% and 30%, amounts of UFA particles destroyed the integrity of ettringite structure, it caused the strength of the HWBM decreased sharply. Therefore, in this work, the UFA dosages of 15% is reasonable in view of utilizing of fly ash and mechanical properties of the HWBM.

### Degree of crystallinity of the HWBM

**Figure 13:**
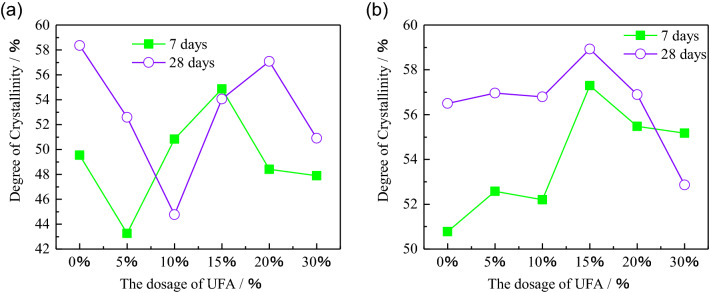
The degree of crystallinity in the HWBM blend with different dosages of UFA. (**a**) Cured at the standard curing condition; (**b**) cured at the laboratory air condition.

Due to the degree of crystallinity have a significant effect on mechanical properties of materials, we calculated the degree of crystallinity of samples according to the XRD pattern for further analyzing the effect mechanism of fly ash on mechanical properties of the HWBM as shown in Fig. 13. Firstly, the main crystal phase is ettringite in the HWBM, so the content of ettringite can be quantified in a degree by the degree of crystallinity. Figure 13a displays the degree of crystallinity of samples cured at the standard curing condition, it can be observed that the degree of crystallinity of HWBM increased with curing time increasing, which indicates that the relative content of ettringite increased with the curing time increasing. After blended with UFA, the degree of crystallinity increased when the UFA dosages were less than 15%, it indicates that a small number of UFA can accelerate the hydration process of the HWBM at the early ages. Whereas the addition of UFA limited the hydration process of later ages.

On the other hand, when the samples were cured at the laboratory air condition, the degree of crystallinity decreased compared with the samples cured at the standard curing condition, which indicates that the formation of ettringite were delayed due to the loss of free water and carbonation of ettringite. Whereas, after adding UFA, the crystallinity of the HWBM first increased and then decreased with the UFA dosages increasing, it indicates that the UFA can improve the weathering resistance of the HWBM when the UFA dosages did not exceed 15%. This result is in conformity with the peak strength as shown in Fig. 8a.

### Effect of UFA on the microstructure of HWBM

#### Curing at the standard curing condition

For observing the hydration products and its morphology of the HWBM with different UFA dosages cured at the standard curing condition, the SEM images of the HWBM samples are given after curing 28 days.

**Figure 14:**
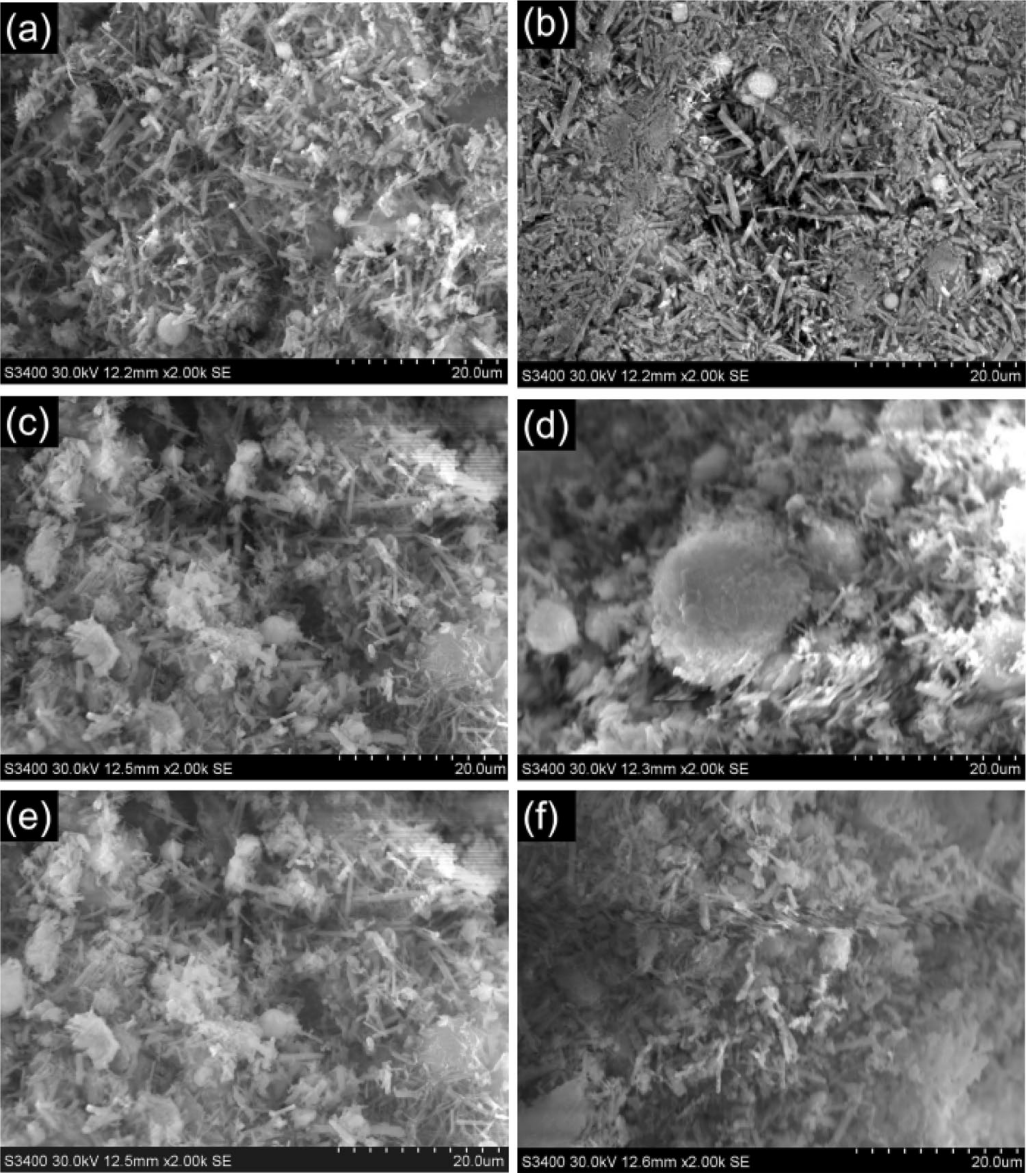
The SEM images of the HWBM blended with different UFA content cured for 28 days curing at the standard condition. (**a**) Control sample; (**b**) 5%; (**c**) 10%; (**d**) 15%; (**e**) 20%; (**f**) 30%.

Figure 14 shows the microstructure of the HWBM with different dosages of UFA at the ages of 28 days under the standard curing condition, it can be observed that the main hydration products of initial HWBM are ettringite and C–S–H as shown in Fig. 14a. The formation of network skeleton structure which consisted of needle-like ettringite is the main source of mechanical properties of the HBWM. But, with the curing time increasing under the standard curing condition, there are a few micro-pore and cracks in the pure HWBM, which may be the result of ettringite formation and expansion in limit space, it caused the failure of an intact structure and the decreasing of the mechanical properties at the 28 days.

Compared with the pure HWBM, the morphology of the HWBM is significant different after adding UFA. It can be found that the content of sheet and cotton-shaped C–S–H gel increased with the UFA dosages increasing as shown in Fig. 14b–f), these gels improved the cohesive force of the ettringite structure, which is beneficial for the later mechanical properties at the ages of 28 days. Additionally, the products of pozzolanic reaction between fly ash and Ca(OH)_2_ also enhanced the residual strength and compressive ability of the HWBM as shown in Fig. 7.

Additionally, the structure of the HWBM became denser when the UFA dosage was 5%, and there are a few globular vitreous particles adhere to the surface of network skeleton structure as shown in Fig. 14b, which indicates that the UFA particles also played a filler role in the HWBM. Thus, when the UFA dosages were less than 20%, the products of pozzolanic reaction improved the cohesion of the samples, and the UFA particles filled in the pores and cracks of hydration products of the HWBM, which caused the mechanical properties parameters increased. However, a amount of UFA particles will lead the dilution of the hydration products and failure of entire structure as shown in Fig. 14f, which caused the strength of the HWBM obviously decreased.

#### Curing at the laboratory air condition

**Figure 15:**
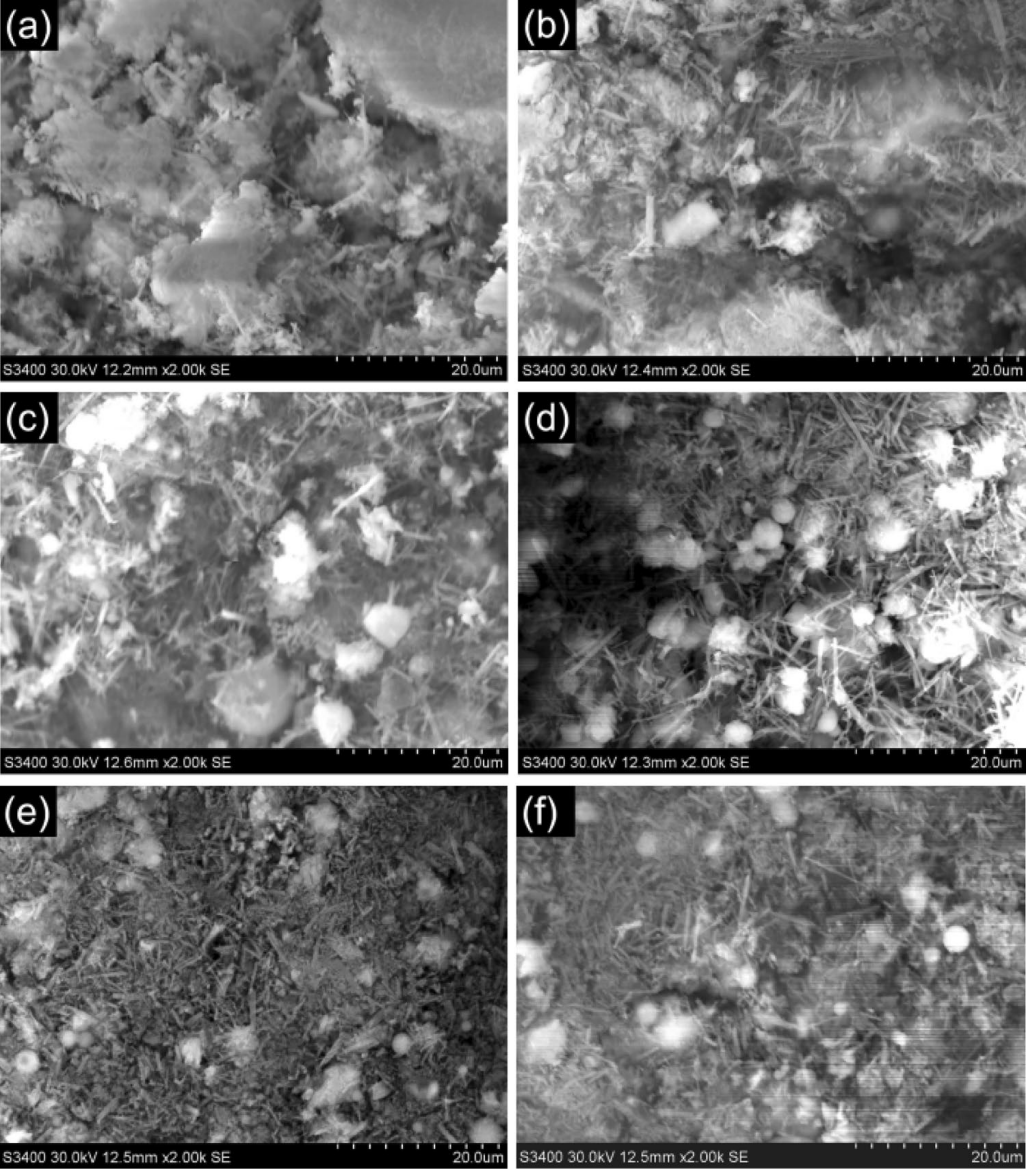
The SEM images of the HWBM blended with different UFA content cured for 28 days curing at the laboratory air condition. (**a**) Control sample; (**b**) 5%; (**c**) 10%; (**d**) 15%; (**e**) 20%; (**f**) 30%.

Under the laboratory air condition, there would be different hydration reactions occurring in the interior of HWBM in comparison to the standard curing condition, such as evaporation of internal free water and carbonation of ettringite, which affects the hydration process and microstructure of the HWBM. Thus, the SEM images of HWBM with different dosages of UFA were displayed in Fig. 15.

As shown in Fig. 15a, it can be observed that the content of ettringite in the HWBM decreased significantly under the laboratory air condition compared with the standard curing condition, while the content of calcite increased. It indicates that the hydration products of the HWBM occurred the weathering carbonation reaction with the CO_2_ in the air, and the formation of the calcite weakened the adhesive property of the HWBM, which make its strength reduced rapidly at the later curing ages. In addition, the evaporation of free water accelerated the weathering carbonation of the hydration products, lead the strength of samples decreased at the 28 days.

However, the adding of UFA filled in the cracks of ettringite and C–S–H delayed the carbonation reaction of the hydration products due to the adhering of UFA particles lead the decreasing of the contact area between hydration products and the CO_2_ in the air, while there is no reaction between the glass-globular fly ash particles and the CO_2_ as shown in Fig. 15b–f. Nevertheless, due to the UFA particles have no adhesive property, the adding of UFA just play a filler role in the HWBM. On the one hand, the filling of UFA particles caused the decreasing of the weathering carbonation in the HWBM, further delayed the decreasing of its strength at the 28 days when the UFA dosages were less than 15%. On the other hand, as shown in Fig. 15e and f, amounts of fly ash particles adhered to the hydration products of the HWBM, which there was a dilution effect on the its microstructure, further destroyed the whole structure of the HWBM, finally lead the obvious decreasing of the strength of the HWBM as shown in Fig. 8a.

In summary, according to the analysis about the effects of UFA on the microstructure of the HWBM, we suggest that the UFA dosages should be no more than 15%, this result is agreed with the XRD analyze as mentioned in the sections “Mechanical properties of the HWBMs blended with UFA” and “Effect of UFA on the hydration products of the HWBM”.

## Discussions

In this work, according to the mentioned in sections “Mechanical properties of the HWBMs blended with UFA”–“Degree of crystallinity of the HWBM”, the pozzolanic reaction occurred among CH_2_, activity SiO_2_ and Al_2_O_3_ in the UFA, and H_2_O, then the formation of more C–S–H gel leaded to the higher compression strength of the HWBM at the later ages (28 days). Meanwhile, the increasing of gel with caking property improved the cohesiveness of the HWBM samples, caused the HWBM to have a higher post-peak strength and compressive ability. Additionally, ultra-fine fly ash has better fineness and activity compared with general fly ash, so it is possible to consume more CH_2_ in the HWBM, which can support the increasing of the strength at the later ages. However, an amount of UFA have not participated in the pozzolanic reaction with the increasing of UFA dosage due to the limitation of CH_2_ content, so they only played the role of finer filler materials in the HWBM, and too much UFA (more than 30% wt.) will cause obvious decrease in the strength of the HWBM. Thus, the dosages of UFA in the HWBM should not exceed 20%.

Additionally, when the samples were cured at the laboratory air condition, the addition of UFA has two main effects on the mechanical properties of the HWBM at the early ages: (a) filler and (b) dilution effect, which the adding of UFA contributed to the increasing of mechanical properties at the later ages (28 days) as a filler materials in the HWBM while the UFA dosages were less than 20%. But when the UFA dosages exceeded 20%, the dilution effect played a more important role and leaded to the mechanical properties of the HWBM decrease significantly. Meanwhile, the evaporation of free water content accelerated early hydration process of the HWBM, which caused the rapid increasing of early strength before the ages of 7 days. But the porosity increasing with the rapid decreasing of free water leaded to the increasing of contact area between the hydration products and air, which caused the weathering carbonation of ettringite and C–S–H. finally lead to the decreasing of the mechanical properties of the HWBM. Thus, the adding of the right amount of UFA can effectively delay the weathering of the HWBM as a finer filler material.

It is notable that the pozzolanic activity of active UFA particles also come into play, which the pozzolanic reaction occurred among on the CaO, Al_2_O_3_ and water, formation of C–S–H and C–A–H further improving the carbonation resistance of HWBM at the later ages. In summary, the adding of UFA can retard the weathering process of the HWBM, further promote the development of strength in the HWBM at the later ages. However, the UFA activity has not been motivated absolutely, due to the limitation of CH_2_ content in the HWBM, which leads to the main effects of UFA on the performance of the HWBM being filler and dilution, then caused the strength of the HWBM decreased rapidly when the UFA dosages were too much.

Therefore, for further improving the reaction activity of the fly ash and promoting its application in mining backfill, amounts of experiments is necessary to enhance the replacement dosage of UFA in the HWBM and ensure the mechanical properties of the HWBM to be improved. Thus, it is necessary to investigate the effect of the proportion of FA and CSA on mechanical properties of the HWBM for improving its performance, and the further investigation about the effects mechanism of the hydration process, hydration products and their proportion, microstructure and interaction on the mechanical properties of the HWBM also is vital for development of the efficient utilization of the fly ash in the mining backfill field.

## Conclusions

The present study investigated the change in mechanical properties and hydration products of the HWBM with the water binder ratio of 2.5:1 after blended with UFA, and the influence of UFA on hydration process and carbonation mechanism under natural environment were analyzed. Therefore, the main findings of this study are given herein.

1. When the samples were cured at the standard curing condition, the strength of HWBMs decreased with the increasing of the UFA contents, especially as the dosage of UFA was 30% by mass. However, at the ages of 28 days, the strength of the HWBM blended with UFA increased while the pure HWBM decreased. It indicates that the UFA can improve the later strength down of the HWBM as the dosages were less than 20%.
2. When the samples were cured at the laboratory air condition, the strength of the HWBM blended with UFA increased at the ages of 28 days as the UFA dosages were less than 20%. Compared with previous illustrates, the ultra-fine fly ash has higher dosages on the HWBM, and the UFA can improve the carbonation resistance of HWBM.
3. The adding of UFA can enhance the residual strength and compressive ability of the HWBM with the water binder ratio of 2.5:1 Which is beneficial for the engineering application of HWBM in backfill mining, and further promoted utilize ratio of FA. But the UFA dosages should not exceed 20% by mass.
4. According to the XRD and SEM results, the adding of UFA can promote the hydration process due to the pozzolanic reaction between the fly ash and CH_2_, which leaded to increasing of mechanical properties of the HWBM at the later ages. Meanwhile, the adding of UFA can enhance the degree of crystallinity of hydration products in HWBM, and further enhance its residual strength and carbonation resistance. Additionally, the main effects of UFA on microstructure of the HWBM were filler and dilution at the early ages.
5. This investigation proves that the UFA have a potential application on the mining HWBM backfill. However, there still are many experiments need to be performed to motivate the activity of UFA and improve the performance of the HWBM blended with UFA.

## Data Availability

The datasets used and/or analyzed during the current study available from the first or corresponding author on reasonable request.
